# Genetic variation in stromal proteins decorin and lumican with breast cancer: investigations in two case-control studies

**DOI:** 10.1186/bcr2201

**Published:** 2008-11-26

**Authors:** Linda E Kelemen, Fergus J Couch, Shahana Ahmed, Alison M Dunning, Paul DP Pharoah, Douglas F Easton, Zachary S Fredericksen, Robert A Vierkant, V Shane Pankratz, Ellen L Goode, Christopher G Scott, David N Rider, Xianshu Wang, James R Cerhan, Celine M Vachon

**Affiliations:** 1 Department of Population Health Research, Alberta Cancer Board, 1331 29th Street NW, Calgary, AB T2N 4N2, Canada; 2 Department of Laboratory Medicine and Pathology, Mayo Clinic College of Medicine, 200 First Street SW, Rochester, MN 55905, USA; 3 Cancer Research UK, Department of Oncology, Strangeways Research Laboratory, University of Cambridge, Worts Causeway, Cambridge CB1 8RN, UK; 4 Department of Public Health and Primary Care, Strangeways Research Laboratory, University of Cambridge, Wort's Causeway, Cambridge CB1 8RN, UK; 5 Department of Health Sciences Research, Mayo Clinic College of Medicine, 200 First Street SW, Rochester, MN 55905, USA

## Abstract

**Introduction:**

The stroma is the supportive framework of biologic tissue in the breast, consisting of various proteins such as the proteoglycans, decorin and lumican. Altered expression of decorin and lumican is associated with breast tumors. We hypothesized that genetic variation in the decorin (*DCN*) and lumican (*LUM*) genes may contribute to breast cancer.

**Methods:**

We investigated associations of 14 common polymorphisms in the *DCN *and *LUM *genes with 798 breast cancer cases and 843 controls from Mayo Clinic, MN, USA. One polymorphism per gene with the strongest risk association in the Mayo Clinic sample was genotyped in 4,470 breast cancer cases and 4,560 controls from East Anglia, England (Studies of Epidemiology and Risk Factors in Cancer Heredity (SEARCH)).

**Results:**

In the Mayo Clinic sample, six polymorphisms were associated with breast cancer risk (*P*_trend _≤ 0.05). The association with *LUM *rs2268578, evaluated further in SEARCH, was positive, although the odds ratios (OR) were weaker and not statistically significant. ORs were 1.4 (95% confidence interval [CI], 1.1 to 1.8) for heterozygotes and 2.2 (95% CI, 1.1 to 4.3; *P*_2 df _= 0.002) for homozygotes in the Mayo Clinic sample, and were 1.1 (95% CI, 0.9 to 1.2) for heterozygotes and 1.4 (95% CI, 1.0 to 2.1; *P*_2 df _= 0.13) for homozygotes in the SEARCH sample. In combined analyses, the ORs were 1.1 (95% CI, 1.0 to 1.2) for heterozygotes and 1.6 (95% CI, 1.2 to 2.3; *P*_2 df _= 0.005) for homozygotes. Positive associations for this polymorphism were observed for estrogen receptor-positive tumors in both the Mayo Clinic sample (OR for heterozygotes = 1.5, 1.1 to 1.9 and OR for homozygotes = 2.5, 1.2 to 5.3;*P*_2 df _= 0.001) and the SEARCH sample (OR for heterozygotes = 1.0, 0.9 to 1.1 and OR for homozygotes = 1.6, 1.0 to 2.5; *P*_2 df _= 0.10). In combined analyses, the ORs were 1.1 (95% CI, 0.9 to 1.2) for heterozygotes and 1.9 (95% CI, 1.3 to 2.8; *P*_2 df _= 0.001) for homozygotes.

**Conclusions:**

Although *LUM *rs2268578 was associated with breast cancer in the Mayo Clinic study, particularly estrogen receptor-positive breast cancer, weaker and modest associations were observed in the SEARCH sample. These modest associations will require larger samples to adequately assess the importance of this polymorphism in breast cancer.

## Introduction

Stromal changes are well documented in breast tumors [1,2] and in preinvasive breast lesions [2,3], and are hypothesized to play a role in breast cancer. The stroma may lead to morphologic changes that manifest as tumors through a stromal reaction or perturbation of epithelium [4] or, conversely, may play an initial landscaping role in tumorigenesis independent of epithelial neoplastic alterations [5].

The stroma is the supportive framework of biologic tissue consisting of an extensive extracellular matrix that supports cells, separates tissues and regulates intercellular communication. The extracellular matrix is composed of different proteins: decorin and lumican are members of the small leucine-rich proteoglycan family of proteins and are involved in matrix assembly and structure, and in the control of cell proliferation [6]. Knockout mice deficient for either the decorin (*DCN*) gene or the lumican (*LUM*) gene are viable, but show skin fragility, marked reductions in tensile strength and loosely packed collagen fibers with abnormal sheath diameters [7,8] – implicating the small leucine-rich proteoglycans as major regulators of collagen fibril assembly that probably play a role in the development of a barrier against cell penetration and infiltration of macromolecules [6].

Recent evidence supports an anti-oncogenic role for decorin. Injection of decorin protein into mammary carcinoma rodent models resulted in a marked reduction in both primary tumor growth and metastatic spread compared with animals injected with vehicle alone [9]. Low levels of decorin protein in invasive breast cancers have also been associated with larger tumor size, shortened duration to progression and poor outcome [10]. The role of lumican in carcinogenesis has been less well studied. Immunohistochemical analyses of breast tissue detected significantly higher lumican protein expression in tumors than in adjacent normal tissue, yet significantly lower decorin protein expression in tumors than in normal breast tissue [11]. Positive associations between decorin and lumican protein expression and mammographic density, a major risk factor for breast cancer, have also been observed [12]. These findings suggest that expression and/or activity of members of the small leucine-rich proteoglycan family may affect breast cancer risk.

Given the role of the stroma in breast cancer [1,2], the occurrence of genetic alterations in the stroma of breast tumors [13-15] and the putative contribution of decorin and lumican to this disease [10,11], we hypothesized, *a priori*, that common genetic variation in the *DCN *and *LUM *genes is associated with breast cancer risk. We evaluated this hypothesis using data from a clinic-based case-control study of breast cancer, with follow-up in a large British case-control study.

## Materials and methods

### Mayo Clinic study design and population

The Mayo Clinic Breast Cancer study is an Institutional Review Board-approved, ongoing clinic-based case-control study initiated in February 2001 at Mayo Clinic, Rochester, MN, USA. The study design has been presented previously [16,17]. Clinic attendance formed the sampling frame for Mayo Clinic cases and controls.

Consecutive cases were women aged 18 years or older with histologically confirmed primary invasive breast carcinoma who were recruited within 6 months of their date of diagnosis. Women with a history of cancer (excluding nonmelanoma skin cancer) were ineligible. Cases lived in the six-state region that defines Mayo Clinic's primary service population (Minnesota, Iowa, Wisconsin, Illinois, North Dakota and South Dakota). Although Mayo Clinic is widely perceived to be a specialty tertiary care facility, it also provides primary care for over 500,000 individuals per year.

Control individuals without prior history of cancer (other than nonmelanoma skin cancer) were frequency matched on age (5-year age category), race and six-state region of residence to cases. Controls were recruited from the outpatient practice of the Divisions of General Internal Medicine and Primary Care Internal Medicine at Mayo Clinic, where they were seen for routine medical examinations.

Written informed consent was obtained from all participants. Case participation was 69% and control participation was 71%. The present investigation genotyped Caucasian women (99% of study participants) enrolled up to 30 June 2005, representing 798 cases and 843 controls.

Both the cases and controls completed a self-administered questionnaire comprised of known or suspected breast cancer risk factors, and they provided blood samples from which genomic DNA was isolated using the Gentra AutoPure LS Purgene salting out methodology (Gentra, Minneapolis, MN, USA). Quantities of 250 ηg genomic DNA were adjusted to 50 ηg/μl before genotyping and were verified using the PicoGreen dsDNA quantitation kit (Molecular Probes, Inc., Eugene, OR, USA). The samples were bar coded to ensure accurate and reliable sample processing and storage.

### SNP selection, genotyping and quality control

All SNPs in the *DCN *and *LUM *genes within 5 kb of the largest cDNA isoform (genome build 35) were selected from the Caucasian samples within the HapMap Consortium's release 21 [18]. We applied the ldSelect program [19] to bin SNPs with minor allele frequency (MAF) ≥ 0.05 and pairwise linkage disequilibrium threshold of *r*^2 ^≥ 0.80. tagSNPs were selected from these bins that met the criteria for predicted likelihood of successful genotyping using the Illumina GoldenGate Assay™ quality score metrics (Illumina Corporation, San Diego, CA, USA). We also included all putative functional SNPs (within 1 kb upstream, 5' UTR, 3' UTR or nonsynonymous) with MAF ≥ 0.05 identified in Ensembl version 34 (Ensembl, European Bioinformatics Institute/Wellcome Trust Sanger Institute, Wellcome Trust Genome Campus, Hinxton, Cambridgeshire, UK). Eight SNPs in *DCN *(including six functional SNPs) and six SNPs in *LUM *(including three functional SNPs) were identified and examined in the Mayo Clinic study based on these two methods of selection.

The *DCN *and *LUM *SNPs were assayed at Illumina Corporation (San Diego, CA, USA) using the GoldenGate Assay™ on the Illumina BeadLab [20-22] as part of a larger Mayo Clinic genetic association study. Successful genotyping was achieved for all 14 selected SNPs in the 798 cases and 843 controls. All but one of the 14 SNPs had MAF ≥ 0.05 among the Mayo controls (Table 1). Concordance between 100 duplicate samples was >99.99% for all assays.

**Table 1:** Genetic polymorphisms in the decorin (*DCN*) and lumican (*LUM*) genes and minor allele frequencies (MAFs) among 843 Caucasian controls, Mayo Clinic 2001 to 2005

Gene	Location	rsID	Polymorphic region	Amino acid change	MAF (controls)
*DCN*	12q21.33	rs7441	Ex8 G/A 3' UTR		0.06
		rs3138268	Ex7 G/A	Met268Thr	0
		rs516115	IVS3 A/G		0.26
		rs3138165^a^	IVS1 G/A 5' UTR		0.06
		rs2070985^a^	IVS1 G/C 5' UTR		0.06
		rs741212	IVS1 A/G 5' UTR		0.12
		rs13312816^a^	IVS1 A/T		0.06
		rs10492230	G/A 5' upstream		0.16
*LUM*	12q21.3–q22	rs1920790	A/C 3' downstream		0.12
		rs17714469	G/A 3' downstream		0.10
		rs10745553	IVS2 G/C		0.15
		rs2268578	IVS2 G/A		0.11
		rs10859110	IVS1 G/A		0.22
		rs17018765	A/G 5' upstream		0.06

^a^Linkage disequilibrium *r*^2 ^≥ 0.98 for all pairwise combinations of these SNPs.

### SEARCH replication study population

Studies of Epidemiology and Risk Factors in Cancer Heredity (SEARCH), an ongoing population-based study of cases with invasive breast cancer ascertained through the Eastern Cancer Registration and Information Centre in England [23], was used as a second, independent study to evaluate significant breast cancer findings in the Mayo Clinic study. All women diagnosed after 1990 in the East Anglia region (median age = 51 years, range = 25 to 69 years) were eligible, with approximately 65% of eligible breast cancer cases enrolled. Unaffected female controls (median age = 65, range = 45 to 81 years) from the same geographic region were randomly selected from the European Prospective Investigation into Cancer and Nutrition -Norfolk component of the European Prospective Investigation into Cancer and Nutrition in East Anglia, with 41% participation. Over 98% of cases and controls were white Europeans. The SEARCH study has been used extensively to evaluate associations between breast cancer risk and SNPs in various genes [23] and as part of a genome-wide association study for breast cancer [16].

Evaluation of the Mayo Clinic findings for *DCN *rs3138165 and *LUM *rs2268578, the SNPs with the most significant associations with risk in each gene, was performed in the SEARCH study. These SNPs were selected for their strength of associations (point estimates and number of cases with two copies of the minor allele) with breast cancer risk to genotype in the SEARCH study comprising 4,470 cases and 4,560 controls with a 5' nuclease assay (Taqman^®^) using the ABI PRISM 7900 HT Sequence Detection System according to manufacturer's instructions (Applied Biosystems, Foster City, CA, USA). Primers and probes were supplied directly by Applied Biosystems [24] as Assays-by-Design™. Successful genotyping was achieved for 98.6% of DNA samples.

### Statistical analysis

Genotypes from the Mayo Clinic cases and controls were used to estimate allele frequencies. Among control subjects only, the genotypes were compared with those expected under Hardy-Weinberg equilibrium using a Pearson goodness-of-fit test; no departures were found.

Pairwise linkage disequilibrium between SNPs was estimated with *r*^2 ^values [25] using Haploview [26]. Individual SNP associations for breast cancer risk were assessed using unconditional logistic regression to estimate the odds ratios (ORs) and 95% confidence intervals (CIs). Analyses compared women with one copy and two copies of the minor allele with women with no copies using a two-degrees-of-freedom (2 df) model. We then assessed the dose-response effect of the SNP using an ordinal (log-additive) model.

Haplotype analyses were performed to identify whether the association with breast cancer risk could be informed further by phased combinations of alleles within each gene. Such combinations of alleles on a single chromosome can provide information about the possible presence of nearby breast cancer risk alleles that were not genotyped. Haplotype frequencies for each gene were estimated using all SNPs within the gene, and associations between individual haplotypes and breast cancer risk were evaluated compared with all other haplotypes combined. Haplotype frequencies for each gene were estimated using all SNPs within the gene, and a global haplotype score test of no association between haplotypes and breast cancer risk was evaluated at the gene level by the method proposed by Schaid and colleagues [27]. When the global haplotype score test suggested significance at the gene level, individual haplotype-specific associations for risk of breast cancer were compared with all other haplotypes combined.

In the SEARCH study, age-adjusted single-SNP associations were conducted using unconditional logistic regression under one-copy/two-copy and ordinal genetic models as described above.

In secondary analyses in both the Mayo Clinic and SEARCH studies, we stratified the data to evaluate the risk of breast cancer attributable to *LUM *rs2268578 in cases with estrogen receptor (ER)-positive and ER-negative tumors compared with control subjects because of recent reports of differential lumican protein expression by tumor ER status [10,28].

Analyses were implemented using Haplo.stats [29], the SAS software system (version 8, 1999; SAS Institute, Cary, NC, USA) and the S-Plus software system (version 7.05, 2005; Insightful Corp., Seattle, WA, USA). Given prior hypotheses that SNPs in the *DCN *and *LUM *genes are associated with breast cancer risk, corrections for multiple testing were not performed. Two-tailed *P *≤ 0.05 was considered statistically significant.

## Results

**Figure 1:**
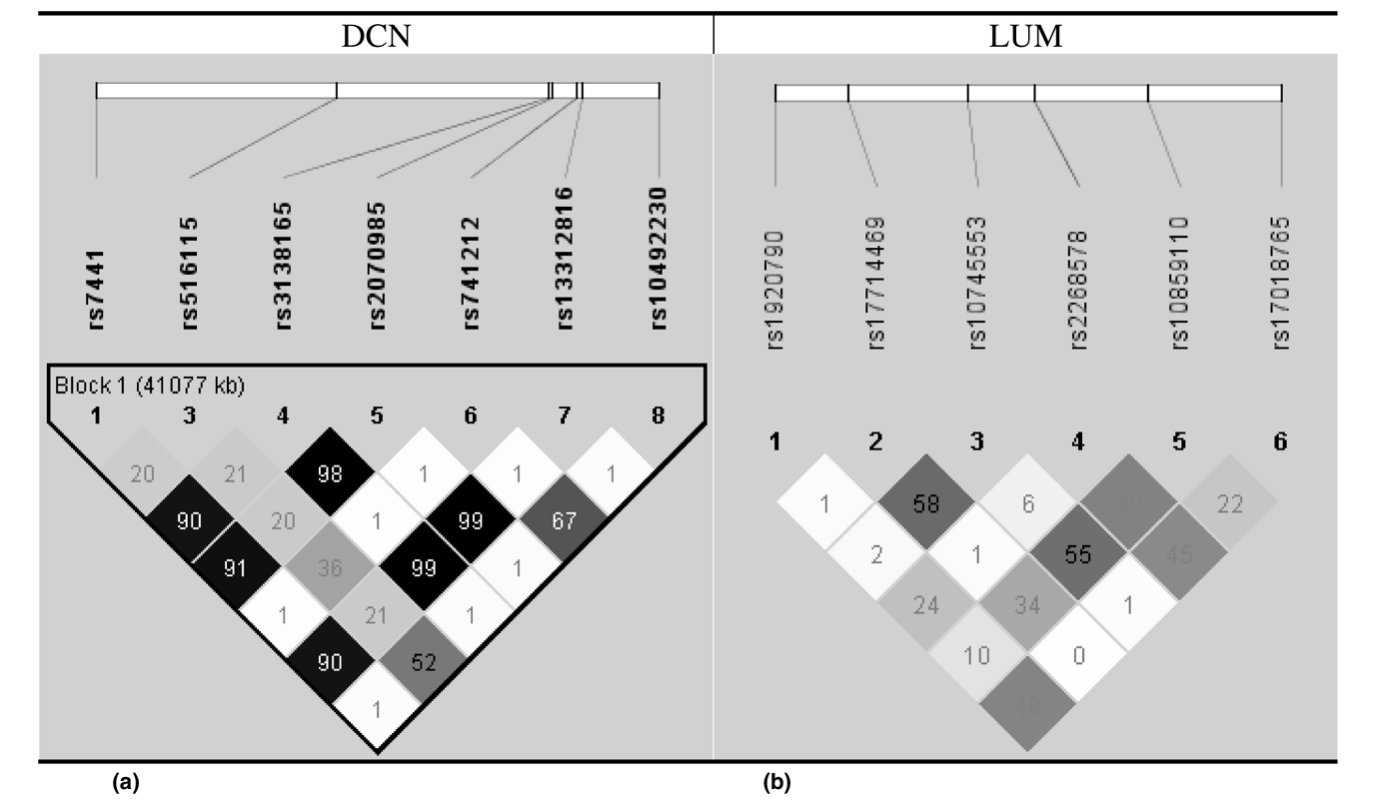
**Linkage disequilibrium plots for polymorphisms in the *DCN *and *LUM *genes**. Linkage disequilibrium (LD) plots for polymorphisms in the Mayo Clinic sample, 2001 to 2005, for **(a) **the decorin (*DCN*) gene and **(b) **the lumican (*LUM*) gene. Shaded regions indicate strength of LD between pairwise combinations of polymorphisms (white, *r*^2 ^= low LD; black, *r*^2 ^= near-perfect LD). Numbers in squares indicate estimates of the pairwise *r*^2^. *DCN *SNP rs3138268 had minor allele frequency = 0 (absent in figure), and *DCN *SNPs rs3138165, rs2070985 and rs13312816 had pairwise *r*^2 ^≥ 0.98: only rs3138165 was included in the statistical analyses.

Eight tagSNPs in *DCN *representing 21 individual SNPs and six tagSNPs in *LUM *representing 15 individual SNPs were identified from the HapMap (Table 1 and Figure 1). Of eight tagSNPs genotyped in *DCN*, three were highly correlated (rs3138165, rs2070985 and rs13312816: pairwise *r*^2 ^≥ 0.98). This redundancy resulted from genotyping all putative functional SNPs in addition to the tagSNPs; only data from one *DCN *tagSNP (rs3138165) are therefore shown in subsequent analyses. Further, another *DCN *tagSNP (rs3138268) had MAF = 0 among control subjects and was excluded from further analyses.

The Mayo Clinic cases and controls were adequately matched on age and region of residence, but differed in distribution by menopausal status, postmenopausal hormone use, age at menarche and smoking (Table 2). Covariates were evaluated for confounding in statistical models, and there was no appreciable difference in risk estimates of association when these covariates were excluded. The results for risk models are therefore presented adjusted for age and region of residence.

**Table 2:** Demographic, personal and lifestyle characteristics among 1,641 Caucasian breast cancer cases and controls, Mayo Clinic 2001 to 2005

		Cases (*n *= 798)		Controls (*n *= 843)	
			
Characteristic	Level	*n*	%^a^	*n*	%
Age (years)	20 to 39	56	7	48	6
	40 to 49	192	24	166	20
	50 to 59	224	28	274	32
	60 to 69	195	24	207	25
	70+	131	16	148	18

State of residence	Minnesota	502	63	552	66
	Wisconsin	69	9	78	9
	Iowa	128	16	147	17
	North Dakota/South Dakota	52	6	41	5
	Illinois	47	6	25	3

Body mass index (kg/m^2^)	Mean (standard deviation)	28	8	27	8

Family history^b^	Yes	366	47	345	43

Postmenopausal status	Yes	480	64	579	72

Age at menarche (years)	<12	132	18	122	16
	12	224	31	184	24
	13	218	30	238	32
	≥ 14	154	21	209	28

Oral contraceptive use	0 months	266	35	243	31
	1 to 48 months	185	24	189	24
	48+ months	310	41	345	44

Postmenopausal hormone use	0 months	430	58	366	49
	1 to 60 months	131	18	160	22
	60+ months	184	25	216	29

Age at first birth (years)	Nulliparous	97	13	128	16
	≤ 20 years	173	22	158	20
	>20 years	501	65	514	64

Smoking (pack years)	None	467	63	500	66
	≤ 4 years	46	6	67	9
	>4 years	231	31	192	25

Alcohol consumption	Never	92	12	94	12
	Monthly	332	43	327	41
	Weekly	259	34	299	37
	Daily	83	11	79	10

^a^Percentages may not total 100 due to missing data. ^b^Family history in first-degree or second-degree relative with breast cancer or ovarian cancer.

### Breast cancer risk analyses

In the individual SNP analyses, three SNPs in *DCN *and three SNPs in *LUM *were associated with breast cancer risk in the Mayo Clinic sample: results of the one-copy/two-copy genetic model and of the ordinal model are presented in Table 3. The ORs associated with each copy of the minor allele varied slightly for *DCN *rs7441 (OR = 1.3, 95% CI = 1.0 to 1.7; *P*_trend _= 0.05), *DCN *rs516115 (OR = 1.2, 95% CI = 1.0 to 1.4; *P*_trend _= 0.03) and *DCN *rs3138165 (OR = 1.3, 95% CI = 1.0 to 1.8; *P*_trend _= 0.03). In *LUM*, the associations with each copy of the minor allele were similar for *LUM *rs2268578 (OR = 1.4, 95% CI = 1.2 to 1.8; *P*_trend _= 0.0003), *LUM *rs10859110 (OR = 1.3, 95% CI = 1.1 to 1.5; *P*_trend _= 0.001) and *LUM *rs17018765 (OR = 1.3, 95% CI = 1.0 to 1.7; *P*_trend _= 0.04).

**Table 3:** Odds ratios (OR) and 95% confidence intervals (CI)^a ^between polymorphisms in the decorin (*DCN*) and lumican (*LUM*) genes and breast cancer risk among 1,641 Caucasian subjects^b^, Mayo Clinic 2001 to 2005

Polymorphism/rsID	MAF	Homozygotes common allele (referent OR = 1)		Heterozygotes^c^			Homozygotes rare allele^c^			Per allele	*P *trend
						
		Cases	Controls	Cases	Controls	OR (95% CI)	Cases	Controls	OR (95% CI)	OR (95% CI)	
*DCN*											
rs7441	0.06	675	744	119	95	1.3 (1.0 to 1.8)	4	4	1.2 (0.3 to 4.7)	1.3 (1.0 to 1.7)	0.05
rs516115	0.26	397	470	336	316	1.2 (1.0 to 1.5)	65	57	1.3 (0.9 to 2.0)	1.2 (1.0 to 1.4)	0.03
rs3138165	0.06	668	741	123	98	1.4 (1.0 to 1.8)	6	4	1.8 (0.5 to 6.3)	1.3 (1.0 to 1.8)	0.03
rs741212	0.12	616	662	167	167	1.1 (0.8 to 1.4)	15	14	1.2 (0.6 to 2.5)	1.1 (0.9 to 1.3)	0.51
rs10492230	0.16	557	589	216	231	1.0 (0.8 to 1.2)	25	23	1.2 (0.7 to 2.1)	1.0 (0.8 to 1.2)	0.88

*LUM*											
rs1920790	0.12	594	653	188	180	1.1 (0.9 to 1.4)	16	10	1.8 (0.8 to 3.9)	1.2 (0.9 to 1.4)	0.13
rs17714469	0.10	647	683	141	152	1.0 (0.7 to 1.2)	10	8	1.3 (0.5 to 3.3)	1.0 (0.8 to 1.3)	1.0
rs10745553	0.15	559	613	214	212	1.1 (0.9 to 1.4)	24	17	1.6 (0.8 to 3.0)	1.1 (0.9 to 1.4)	0.14
rs2268578	0.11	567	661	207	167	1.4 (1.1 to 1.8)	23	13	2.2 (1.1 to 4.3)^d^	1.4 (1.2 to 1.8)	0.0003
rs10859110	0.22	418	517	331	283	1.4 (1.2 to 1.7)	49	43	1.4 (0.9 to 2.2)^d^	1.3 (1.1 to 1.5)	0.001
rs17018765	0.06	673	744	121	95	1.4 (1.0 to 1.8)	4	4	1.2 (0.3 to 4.7)	1.3 (1.0 to 1.7)	0.04

MAF, minor allele frequency. ^a^Adjusted for age and region of residence (Minnesota, Iowa, Wisconsin, Illinois, North Dakota and South Dakota). ^b^798 cases and 843 controls. ^c^Referent (OR = 1) is homozygous common allele group. ^d^Two-degrees-of-freedom test, *P *= 0.002.

At the gene level, only *LUM *(*P *= 0.01) showed evidence of a significant association across haplotypes for breast cancer risk (Table 4). Of nine haplotypes observed in *LUM*, one six-SNP haplotype (haplotype 2a) – accounting for 71% of all estimated haplotypes – was associated with decreased breast cancer risk (*P *= 0.01), while two separate haplotypes with 6% (haplotype 2 h) and 7% (haplotype 2i) frequency were associated with increased risk (*P *≤ 0.03).

**Table 4:** Gene-level^a ^analysis of the decorin (*DCN*) and lumican (*LUM*) genes with breast cancer risk among 1,641 Caucasian subjects^b^, Mayo Clinic 2001 to 2005

Gene/haplotype	Global haplotype score test *P *value	Estimated haplotype frequency	Individual haplotype score test^c^	Individual haplotype *P *value^d^
*DCN*^e^	0.18			
1a: AAAAC		0.73	-2.19	0.03
1b: AGAAT		0.05	-0.65	0.52
1c: GGAAC		0.001	0.38	0.70
1d: AGAGT		0.12	0.59	0.55
1e: AGGAC		0.004	0.82	0.41
1f: AGAAC		0.03	1.82	0.07
1g: GGGAC		0.07	1.92	0.05

*LUM*^f^	0.01			
2a: AGGGGA		0.71	-2.44	0.01
2b: CGGGGA		0.05	-0.74	0.46
2c: CGGGAA		0.004	-0.04	0.97
2d: AACGAA		0.10	0.42	0.67
2e: AGGGAA		0.003	0.89	0.37
2f: CGGAAA		0.01	1.08	0.28
2g: AGGAAA		0.003	1.38	0.17
2h: AGCAAA		0.06	2.18	0.03
2i: CGGAAG		0.07	2.29	0.02

^a^Adjusted for age and region of residence (Minnesota, Iowa, Wisconsin, Illinois, North Dakota and South Dakota). ^b^798 cases and 843 controls. ^c^Score statistics comparing haplotype of interest with all other haplotypes combined. Negative values imply decreased risk of breast cancer, whereas positive values imply increased risk. ^d^*P *value comparing haplotype of interest with all other haplotypes combined. ^e^Haplotype-forming SNPs in *DCN *are rs7441 (A/G), rs516115 (A/G), rs3138165 (A/G), rs741212 (A/G), rs10492230 (A/G). ^f^Haplotype-forming SNPs in *LUM *are rs1920790 (A/C), rs17714469 (G/A), rs10745553 (G/C), rs2268578 (G/A), rs10859110 (G/A), rs17018765 (A/G).

The *DCN *rs3138165 and *LUM *rs2268578 SNPs were genotyped in the SEARCH study. The association of *DCN *rs3138165 in the Mayo Clinic sample was not confirmed in the SEARCH study, whereas *LUM *rs2268578 was positively associated with breast cancer risk but the ORs attenuated and did not reach statistical significance (Table 5). Compared with women with no copies of the minor allele in *LUM *rs2268578, women with two copies showed the greatest risk for breast cancer in both the Mayo Clinic sample (OR = 2.2, 95% CI = 1.1 to 4.3; *P*_2 df _= 0.002) and the SEARCH study sample (OR = 1.4, 95% CI = 1.0 to 2.1; *P*_2 df _= 0.13). When the data from the two studies were pooled in age-adjusted and study-adjusted models, *LUM *rs2268578 was associated with increased risk among heterozygotes (OR = 1.1, 95% CI = 1.0 to 1.2) and homozygotes (OR = 1.6, 95% CI = 1.2 to 2.3; *P*_2 df _= 0.005) (Table 5). The corresponding per-minor allele risk was 1.1 (95% CI = 1.0 to 1.2; *P*_trend _= 0.004). These data suggest that *LUM *rs2268578 or a variant in strong linkage disequilibrium with rs2268578 may be a risk factor for breast cancer.

**Table 5:** Odds ratios (OR) and 95% confidence intervals (CI)^a ^between polymorphisms in the decorin (*DCN*) and lumican (*LUM*) genes and breast cancer risk among 1,641 Caucasian subjects (Mayo Clinic 2001 to 2005) and 9,030 Caucasian subjects (SEARCH study 1990 2005)^b^

	*DCN *rs3138165					*LUM *rs2268578				
		
Model	Mayo Clinic sample (MAF = 0.06)		SEARCH sample (MAF = 0.07)		Pooled Mayo Clinic + SEARCH sample	Mayo Clinic sample (MAF = 0.11)		SEARCH sample (MAF = 0.12)		Pooled Mayo Clinic + SEARCH sample
						
	Cases/controls	OR (95% CI)	Cases/controls	OR (95% CI)	OR (95% CI)	Cases/controls	OR (95% CI)	Cases/controls	OR (95% CI)	OR (95% CI)
General										
0 copy	668/741	1.0 (referent)	3,801/3,965	1.0 (referent)	1.0 (referent)	567/661	1.0 (referent)	3,306/3,506	1.0 (referent)	1.0 (referent)
1 copy	123/98	1.4 (1.0 to 1.8)	547/558	1.0 (0.9 to 1.2)	1.1 (0.9 to 1.2)	207/167	1.4 (1.1 to 1.8)	972/980	1.1 (0.9 to 1.2)	1.1 (1.0 to 1.2)
2 copies	6/4	1.8 (0.5 to 6.3)	19/20	0.8 (0.4 to 1.7)	1.1 (0.6 to 2.1)	23/13	2.2 (1.1 to 4.3)	85/62	1.4 (1.0 to 2.1)	1.6 (1.2 to 2.3)
*P *value^c^		0.08		0.87	0.61		0.002		0.13	0.005
Ordinal		1.3 (1.0 to 1.8)		1.0 (0.9 to 1.1)	1.1 (0.9 to 1.2)		1.4 (1.2 to 1.8)		1.1 (1.0 to 1.2)	1.1 (1.0 to 1.2)
*P *trend		0.03		0.96	0.32		0.0003		0.10	0.004

MAF, minor allele frequency. ^a^Mayo Clinic sample adjusted for age and region of residence (Minnesota, Iowa, Wisconsin, Illinois, North Dakota and South Dakota); SEARCH sample adjusted for age; pooled Mayo Clinic + SEARCH sample adjusted for age and study. ^b^798 cases and 843 controls (Mayo Clinic sample), and 4,470 cases and 4,560 controls (SEARCH sample). ^c^Two-degrees-of-freedom test.

### Estrogen receptor subgroup analyses

Women with two copies compared with no copies of the minor allele in *LUM *rs2268578 were at higher risk, compared with control individuals, of ER-positive breast cancer in both the Mayo Clinic study (OR = 2.5, 95% CI = 1.2 to 5.3; *P*_2 df _= 0.001) and the SEARCH study (OR = 1.6, 95% CI = 1.0 to 2.5; *P*_2 df _= 0.10) (Table 6). The per-minor allele risks were 1.5 (95% CI = 1.2 to 1.9; *P*_trend _= 0.0003) in the Mayo Clinic sample and 1.1 (95% CI = 0.9 to 1.2; *P*_trend _= 0.29) in the SEARCH sample. In pooled age-adjusted and study-adjusted analyses, *LUM *rs2268578 was associated with ER-positive tumors among heterozygotes (OR = 1.1, 95% CI = 0.9 to 1.2) and homozygotes (OR = 1.9, 95% CI = 1.3 to 2.8; *P*_2 df _= 0.001). The corresponding per-minor allele risk was 1.1 (95% CI = 1.0 to 1.3; *P*_trend _= 0.01).

**Table 6:** Odds ratios (OR) and 95% confidence intervals (CI)^a ^between *LUM *SNP rs2268578 and breast cancer risk, stratified by tumor estrogen receptor status among 1,641 Caucasian subjects (Mayo Clinic 2001 to 2005) and 9,030 Caucasian subjects (SEARCH study 1990 to 2005)^b^

	Estrogen receptor-positive					Estrogen receptor-negative				
		
Model	Mayo Clinic sample		SEARCH sample		Pooled Mayo Clinic + SEARCH sample	Mayo Clinic sample		SEARCH sample		Pooled Mayo Clinic + SEARCH sample
						
	Cases/controls	OR (95% CI)	Cases/controls	OR (95% CI)	OR (95% CI)	Cases/controls	OR (95% CI)	Cases/controls	OR (95% CI)	OR (95% CI)
General										
0 copy	340/661	1.0 (referent)	1,506/3,506	1.0 (referent)	1.0 (referent)	86/661	1.0 (referent)	344/3,506	1.0 (referent)	1.0 (referent)
1 copy	125/167	1.5 (1.1 to 1.9)	431/980	1.0 (0.9 to 1.1)	1.1 (0.9 to 1.2)	20/167	0.9 (0.5 to 1.5)	107/980	1.0 (0.8 to 1.4)	1.0 (0.8 to 1.3)
2 copies	17/13	2.5 (1.2 to 5.3)	47/62	1.6 (1.0 to 2.5)	1.9 (1.3 to 2.8)	3/13	1.7 (0.4 to 6.5)	5/62	0.8 (0.3 to 2.0)	1.0 (0.5 to 2.2)
*P *value^c^		0.001		0.10	0.001		0.67		0.79	0.98
Ordinal		1.5 (1.2 to 1.9)		1.1 (0.9 to 1.2)	1.1 (1.0 to 1.3)		1.0 (0.7 to 1.6)		1.0 (0.8 to 1.3)	1.0 (0.8 to 1.2)
*P *trend		0.0003		0.29	0.01		0.89		0.93	0.86

^a^Mayo sample adjusted for age and region of residence (Minnesota, Iowa, Wisconsin, Illinois, North Dakota and South Dakota); SEARCH sample adjusted for age; pooled Mayo + SEARCH sample adjusted for age and study. ^b^798 cases and 843 controls (Mayo Clinic sample), and 4,470 cases and 4,560 controls (SEARCH sample). Numbers do not total due to missing tumor status: 1,985 (44%) in SEARCH and 206 (26%) in Mayo Clinic sample. ^c^Two-degrees-of-freedom test.

The number of ER-negative cases was small in both samples and associations with breast cancer were not evident (Table 6). These data may suggest that the associations observed from the main effects models in both studies (Table 5) are due to the findings from ER-positive tumors. Almost one-half of SEARCH study tumors and one-quarter of Mayo Clinic tumors, however, could not be classified – precluding a definitive interpretation of the results, particularly for ER-negative breast cancer.

## Discussion

We found a positive association between *LUM *rs2268578 and breast cancer risk in two large independent case-control studies, although the association in the SEARCH sample was attenuated and did not reach statistical significance. Two copies of the minor allele in *LUM *rs2268578 were associated with an average 60% increased risk of breast cancer compared with women with no copies, and the data suggest increased risk for ER-positive tumors. *DCN *rs3138165 was positively associated with breast cancer risk in the Mayo Clinic sample but the finding was not confirmed in the SEARCH sample. Haplotypes from each gene were also associated with breast cancer risk in the Mayo Clinic sample.

The weaker association of *LUM *rs2268578 with breast cancer risk in the SEARCH sample may be due to a lack of causal association of this SNP with breast cancer, or the results may be a more valid estimate of effect. It is not uncommon for replicated findings to report ORs for the variant homozygote that are weaker compared with the initial study's findings, a phenomenon known as winner's curse [30]. Furthermore, a small sample size can frequently result in insufficient power to detect minor contributions of one or more alleles [30] – as we observed with the results from the SEARCH sample.

In *post hoc *evaluation we also compared our results for *LUM *rs2268578 with those from the Cancer Genetic Markers of Susceptibility (CGEMS) genome-wide association study of breast cancer risk among 1,145 postmenopausal breast cancer cases and 1,142 controls of European ancestry from the Nurses' Health Study [31,32]. When restricted to postmenopausal women, the associations with breast cancer risk were weaker but remained positive in the Mayo Clinic and SEARCH samples; however, in the CGEMS data, women with one copy (OR = 1.1) or two copies (OR = 0.9, *P*_2 df _= 0.52), compared with no copies, of the minor allele were not at risk of breast cancer despite similar MAF = 0.12 among controls. The CGEMS data did not report ER-positive or ER-negative results. At MAF = 0.12, if the true OR for homozygotes is in the range 1.1 to 1.5, then a much larger study than SEARCH or CGEMS would be needed to have sufficient power to replicate the association at either the conventional (*P *≤ 0.05) or the genome-wide (*P *≤ 10^-7^) level of significance. It should be noted that, even in the combined Mayo Clinic and SEARCH samples, there were only 108 cases and 75 controls homozygous for the minor allele. Comparable information from CGEMS is not available. It remains possible that the distribution of other exposures – for example, 60% of Mayo Clinic cases did not use postmenopausal hormones versus <30% in the Nurses' Health Study [33] – may also explain the differences in genetic associations with CGEMS.

The hypothesis that genetic variation in *LUM *is associated with breast cancer is based on a recent series of reports by Watson and colleagues of altered regulation of *LUM *in human breast tumors [10,11]. Using *in situ *hybridization and western blot techniques, *LUM *mRNA levels were significantly higher (*P *< 0.0001) in stroma associated with breast carcinoma compared with stroma associated with adjacent normal tissue in the same woman [11]. In the present study, the observed risk associated with breast cancer from the *LUM *intronic SNP rs2268578 or from a SNP in strong linkage disequilibrium with rs2268578 may be consistent with increased protein expression of lumican in the study by Leygue and colleagues [11], if it represents a negative host response contributing to early tumor development through increased proteolysis or altered lumican deposition that precedes disorganized collagenous stroma [11]. Elucidation of the functional impact of the *LUM *SNP(s) is needed in order to provide insight into the effects on risk.

Associations with breast cancer risk for the *DCN *and *LUM *genes were observed with haplotype analyses in the Mayo Clinic sample. Of note, the three haplotypes in *LUM *that were associated with breast cancer risk had in common the G allele at position 2 (rs17714469) and a graduated change in the combination of alleles in the last three positions (rs2268578, rs10859110 and rs17018765) from GGA (haplotype 2a), associated with decreased risk, to AAA (haplotype 2h) and AAG (haplotype 2g), each associated with increased risk. It is possible that the association between breast cancer risk and these inferred haplotypes may be attributable to *LUM *rs2268578 or a SNP in strong linkage disequilibrium with rs2268578 because this single SNP was also associated with risk in both the Mayo Clinic and SEARCH samples. Further association testing in this chromosomal region – based on genotypes from a denser marker set – is required, however, in order to fully understand the nature of the relationship.

The increased risk of breast cancer from *LUM *rs2268578 may be due to the positive association with ER-positive breast tumors in both the Mayo Clinic and SEARCH samples, although the analyses were underpowered and were not based on *a priori *hypotheses. The association between lumican expression and ER-positive breast tumors is supported by the findings from two studies [10,28]. Troup and colleagues [10] found that a greater number of ER-positive tumors (*n *= 99) compared with ER-negative tumors (*n *= 6) had lumican protein expression ≥ 25th percentile among 140 women with breast cancer (*P *= 0.002). Mackay and colleagues [28] evaluated gene microarray expression profiles in biopsies obtained from 34 women with primary ER-positive breast cancer before and after a 2-week intervention of aromatase inhibitor treatment. Among the 2,418 genes with the greatest variability in expression, the *LUM *gene was the most highly upregulated by a factor, on average, of 2.9-fold following aromatase inhibitor treatment, and the *DCN *gene was upregulated by a factor of 2.3. Collectively, these data suggest a potential mechanistic link between *LUM *expression and ER-positive tumors, which requires further investigation.

The strengths of the present study include the incorporation of a second, independent large sample of cases and controls to confirm initial findings. Our study populations were enrolled from defined regions, were of Caucasian ancestry and were less probably influenced by population stratification [34]. This does not necessarily, however, allow generalization of the observed association with breast cancer to other ethnic/racial populations [35]. One limitation is that the classification of tumors by ER status was not centrally reviewed and a large proportion of tumors could not be classified, hampering a strong conclusion of the association of the *LUM *SNP by ER status. Furthermore, it remains possible that the other variants in *DCN *and *LUM *that were not selected for genotyping in SEARCH may also influence breast cancer risk.

## Conclusion

Although *LUM *rs2268578 was associated with breast cancer in the Mayo Clinic study, particularly ER-positive breast cancer, weaker and modest associations were observed in the SEARCH sample. Evaluation of this SNP in a larger study (such as the Breast Cancer Association Consortium) along with functional studies will be needed to adequately assess the importance of this SNP in breast cancer.

## Abbreviations

CGEMS: Cancer Genetic Markers of Susceptibility; *DCN*: decorin gene; 2 df: two degrees of freedom; ER, estrogen receptor; *LUM*: lumican gene; MAF: minor allele frequency; SEARCH, Studies of Epidemiology and Risk Factors in Cancer Heredity; SNP: single nucleotide polymorphism; UTR: untranslated region.

## Competing interests

The authors declare that they have no competing interests.

## Authors' contributions

LEK drafted the manuscript. LEK, FJC and CMV revised the manuscript for intellectual content. CMV conceived the study hypothesis and selected the genes. FJC, VSP, ELG, DNR, XW, JRC and CMV designed the Mayo Clinic study and acquired data. ZSF, RAV, VSP and CGS performed the statistical analyses. FJC and CMV were responsible for funding for the Mayo Clinic Study. AMD, PDPP and DFE were responsible for study design and funding for, and SA for genotyping in, the SEARCH study. All authors contributed to data interpretation, and read and approved the final manuscript.
